# Intra-species diversity of *Clostridium perfringens*: A diverse genetic repertoire reveals its pathogenic potential

**DOI:** 10.3389/fmicb.2022.952081

**Published:** 2022-07-22

**Authors:** Anny Camargo, Enzo Guerrero-Araya, Sergio Castañeda, Laura Vega, María X. Cardenas-Alvarez, César Rodríguez, Daniel Paredes-Sabja, Juan David Ramírez, Marina Muñoz

**Affiliations:** ^1^Centro de Investigaciones en Microbiología y Biotecnología-UR (CIMBIUR), Facultad de Ciencias Naturales, Universidad del Rosario, Bogotá, Colombia; ^2^Faculty of Health Sciences, Universidad de Boyacá, Tunja, Colombia; ^3^ANID, Millennium Science Initiative Program, Millennium Nucleus in the Biology of the Intestinal Microbiota, Santiago, Chile; ^4^Department of Pharmacology, University of North Carolina, Chapel Hill, NC, United States; ^5^Laboratorio de Investigación en Bacteriología Anaerobia, Facultad de Microbiología, Centro de Investigación en Enfermedades Tropicales, Universidad de Costa Rica, San José, Costa Rica; ^6^Department of Biology, Texas A&M University, College Station, TX, United States; ^7^Molecular Microbiology Laboratory, Department of Pathology, Molecular and Cell-Based Medicine, Icahn School of Medicine at Mount Sinai, New York, NY, United States

**Keywords:** *Clostridium perfringens*, intra-species diversity, multilocus sequence typing, genomic epidemiology, toxinotypes

## Abstract

*Clostridium perfringens* is the causative agent of many enterotoxic diseases in humans and animals, and it is present in diverse environments (soil, food, sewage, and water). Multilocus Sequence Typing (MLST) and Whole Genome Sequencing (WGS) have provided a general approach about genetic diversity of *C. perfringens*; however, those studies are limited to specific locations and often include a reduced number of genomes. In this study, 372 *C. perfringens* genomes from multiple locations and sources were used to assess the genetic diversity and phylogenetic relatedness of this pathogen. *In silico* MLST was used for typing the isolates, and the resulting sequence types (ST) were assigned to clonal complexes (CC) based on allelic profiles that differ from its founder by up to double-locus variants. A pangenome analysis was conducted, and a core genome-based phylogenetic tree was created to define phylogenetic groups. Additionally, key virulence factors, toxinotypes, and antibiotic resistance genes were identified using ABRicate against Virulence Factor Database (VFDB), TOXiper, and Resfinder, respectively. The majority of the *C. perfringens* genomes found in publicly available databases were derived from food (*n* = 85) and bird (*n* = 85) isolates. A total of 195 STs, some of them shared between sources such as food and human, horses and dogs, and environment and birds, were grouped in 25 CC and distributed along five phylogenetic groups. Fifty-three percent of the genomes were allocated to toxinotype A, followed by F (32%) and G (7%). The most frequently found virulence factors based on > 70% coverage and 99.95% identity were *plc* (100%), *nanH* (99%), *ccp* (99%), and *colA* (98%), which encode an alpha-toxin, a sialidase, an alpha-clostripain, and a collagenase, respectively, while *tetA* (39.5%) and *tetB* (36.2%), which mediate tetracycline resistance determinants, were the most common antibiotic resistance genes detected. The analyses conducted here showed a better view of the presence of this pathogen across several host species. They also confirm that the genetic diversity of *C. perfringens* is based on a large number of virulence factors that vary among phylogroups, and antibiotic resistance markers, especially to tetracyclines, aminoglycosides, and macrolides. Those characteristics highlight the importance of *C. perfringens* as a one of the most common causes of foodborne illness.

## Introduction

*Clostridium perfringens* is an anaerobic, Gram-positive, spore-forming bacillus capable of surviving extreme conditions such as high temperature (> 60°C) and low nutrient levels (Hassan et al., 2015). In 2002, the first *C. perfringens* genome was sequenced showing 2,660 protein-codifying regions, ten rRNA genes, and a low G + C content (28.6%; Shimizu et al., 2002). Since then, many studies have revealed the presence of genes that codify to multiple virulence factors involved in the pathogenicity of this species (Li and BA, 2014; Revitt-Mills et al., 2015; Kiu et al., 2017; Abdel-Glil et al., 2021), such as the alpha (CPA), beta (CPB), epsilon (ETX), iota (ITX), enterotoxin (CPE), and necrotic B-like (NetB) toxins (Awad et al., 1995; Sarker et al., 1999; Keyburn et al., 2008; Garcia et al., 2013; Rood et al., 2018), that contribute to neurologic, histotoxic, and intestinal infections in animals and humans and are used to classify strains in seven different toxin types (A to G; Kiu and Lindsay, 2018).

Despite the usefulness of toxin typing in epidemiology and diagnosis, Multilocus Sequence Typing (MLST) has been implemented as an alternative approach for *C. perfringens* typing (Chalmers et al., 2008; Deguchi et al., 2009; Verma et al., 2020). This method is based on the presence and combination of internal fragments of eight housekeeping genes, which in *C. perfringens* includes *colA*, *groEL*, *sodA*, *plc*, *gyrB*, *sigK*, *pgk*, and *nadA*. The sequences are assigned as distinct alleles creating a unique allelic profile or sequence type (ST). Using the goeBURST algorithm, the STs are classified into groups of genetically related organisms called clonal complexes (CC), which provide insights about evolution and diversification processes and allow intra-species analyses (Larsen et al., 2012; Uzal et al., 2014; Page et al., 2017) such as epidemiological and phylogenetical associations (Maiden, 2006). In addition, the identification of shared STs allows the assessment of possible transmission routes between hosts, a third shared source, and genetic stability in the lineage (Xiao et al., 2012).

Other methods, like Whole Genome Sequencing (WGS) have enabled a better understanding of bacterial pathogens through a high-resolution characterization of their genetic variation and evolution (Raskin et al., 2006). Recently, a genomic analysis allocated 206 publicly available *C. perfringens* genomes into five phylogroups (I–V) linked to different disease outcomes and hosts. Phylogroup I included human food poisoning strains and phylogroup II mostly grouped isolates from enteric lesions in horses and dogs. Phylogroup III was the most abundant and heterogeneous group, containing a variety of strains from different hosts causing multiple diseases, while phylogroups IV and V were less abundant (Abdel-Glil et al., 2021). Additionally, the use of WGS has allowed the identification of virulence factors such as sialidases and hyaluronidases along with other toxins associated with clinical outcomes (Kiu et al., 2017; Abdel-Glil et al., 2021). Furthermore, over the last few years molecular markers linked to antibiotic resistance to tetracycline, rifamycin, and aminoglycoside among others, have been recognized as a potential risk for treatment of the infections caused by *C. perfringens* (Kiu and Lindsay, 2018).

So far, *C. perfringens* genomic studies have been limited to a few geographic locations or to a small number of genomes. Hence, to better understand the global diversity of *C. perfringens*, we conducted a comparative genome analysis of 372 genomes from multiple locations and sources. Our goal was to determine the intra-species diversity and phylogenetic relationships of *C. perfringens*, as well as to identify and characterize key molecular markers associated with its pathogenicity, virulence, and antibiotic resistance from whole genome analyses.

## Materials and methods

### Strain and genome collection

A total of 372 *C. perfringens* genomes were included in our analysis. Two strains sequenced specifically for this study were recovered from human feces and water, in 2013 and 2019, respectively. Briefly, samples were collected in sterile screw cap containers to avoid direct oxygen exposure (Siah et al., 2014) and were grown on tryptose sulfite cycloserine (TSC) agar (Merck) under anaerobic conditions by using anaerobic jars with anaerobic atmosphere generation pouches (AnaeroGen, Thermo Scientific, Oxoid) at 37°C for 24 h. Genomic DNA was extracted using the Wizard Genomic DNA Purification Kit (Promega). The quantity of the extracted DNA was assessed using a Qubit 2.0 Fluorometer. DNA library preparation and paired-end whole genome sequencing (2 × 150 bp) were conducted on the Illumina NextSeq platform 2000 at the Microbial Genome Sequencing Center (MiGS). The assembled genomes were deposited at DDBJ/ENA/GenBank as part of a Bioproject under the accession number PRJNA836622.

Publicly available sequence data of 370 *C. perfringens* genomes was also included in our study for comparative analyses. These data comprised 197 genome assemblies from the Pathosystems Resource Integration Center (PATRIC; Wattam et al., 2014; accessed on May 20th, 2021), 40 assemblies from the Public databases for molecular typing and microbial genome diversity (PubMLST; Jolley et al., 2018; accessed on May 14th, 2021), and 133 reads that were retrieved from the European Nucleotide Archive (ENA) database^1^ using the keywords “*C. perfringens*” (accessed on Jun 20th, 2021). Metadata and access numbers were verified to avoid duplicates (Supplementary Table S1).

### Taxonomic classification, genome assembly and annotation

Quality stats of reads were collected using FastQC.^2^ The 133 raw read pairs from ENA were processed with Trimmomatic v. 0.38 to remove low-quality bases below *Phred* 30 and adapter sequences (Bolger et al., 2014). *De novo* assembly was conducted using SPAdes v. 3.14.1(Bankevich et al., 2012) with the default settings. The quality of the resulting assemblies was assessed with Quast v5.0.2 (Gurevich et al., 2013).

The taxonomic classification of the assemblies was verified using Kraken v1.1.1 (Wood and Salzberg, 2014) with default parameters and using standard databases. Additionally, average nucleotide identity (ANI; Richter and Rossello-Mora, 2009) was calculated with pyANI.^3^ An average identity percentage above 95% denotes strains that belong to the same species. *C. perfringens* ATCC 13124 was used as a reference strain for this analysis. The assemblies generated by SPAdes and the ones obtained from public databases were annotated with PROKKA v. 1.11 (Seemann, 2014) with the default parameters and using the UniprotKB (SwissProt) database (Research UCJNa, 2017), considering kingdom specific databases for Bacteria. Annotated assemblies were taken to calculate the pangenome using Roary (Page et al., 2015). Genes present in 95% of the genomes with at least 95% of identity were designated as core genes.

### *In silico* MLST assignment

To assign isolates to sequence type (ST), *in silico* MLST was performed using FastMLST v0.0.14^4^ (Guerrero-Araya et al., 2020) with the default parameters. Concatenated sequences of the eight housekeeping genes of the scheme were extracted (*colA*, *groEL*, *sodA*, *plc*, *gyrB*, *sigK*, *pgk*, and *nadA*) and novel STs were submitted to the PubMLST database for identification (accessed on May 14th, 2021). Global optimal eBURST (goeBURST)^5^ was used to visualize CC, defined in this study as closely related STs that differ from a common founder at up to two of the eight loci used in the MLST scheme (DLV, double locus variant; Francisco et al., 2012).

#### Phylogenetic reconstruction based on MLST and core genome gene sequences

Concatenated sequences of the eight housekeeping genes belonging to the MLST scheme were used as input for a multiple alignment. Likewise, core genes were concatenated and aligned using MAFFT v7.407. The best-fit model for base substitution in IQ-TREE v2.0.3 was selected to infer phylogenetic relationships by maximum likelihood (ML) and ultrafast bootstrap (1,000 repetitions; Nguyen et al., 2015). A clade was considered well supported when the bootstrap was ≥ 80%, as previously indicated (Wróbel, 2008; Minh et al., 2013). The resulting trees were visualized and edited using iTOL v.5 (Letunic and Bork, 2021). To confirm the phylogenetic clades generated by IQ-TREE, a NeighbourNet network was reconstructed in SplitsTree4 software v4.17.0 (Huson and Bryant, 2006) from a core genome alignment and producing a splits graph representing sequence distances (Huson, 1998).

Reference genomes were included to classify isolates into phylogenetic groups. For Phylogroup I, C. perfringens Darmbrand NCTC8081 (ERR1656460) was selected, which is responsible for human enteritis Necroticans cases in Germany in the 1940s and is genetically related to strains carrying chromosomal cpe (Lindström et al., 2011; Ma et al., 2012). For Phylogroup II, strains involved in necrotizing enteritis in foals and hemorrhagic gastroenteritis in dogs, and denoted with the prefix JFP were chosen (GCA_001949795.1, GCA_001949805.1, GCA_001949775.1; Abdel-Glil et al., 2021). For Phylogroup III, *C. perfringens* ATCC3626 (GCA_000171155.1) and *C. perfringens* ATCC13124 (GCA_000013285.1) were used, and for Phylogroup IV, *C. perfringens* type D (GCA_006385425.1) isolated from a sheep. Phylogroup V was established based on the strain *C. perfringens* Tumat (GCA_003990265.1), Which was isolated from the mummified remains of an ancient puppy found in Siberian Permafrost (Abdel-Glil et al., 2021).

### *In silico* identification of virulence and antimicrobial resistance genes

Toxin detection (CPA, CPB, ETX, IAP, CPE, and *netB*) and typing of the 372 isolates were conducted with ABRicate v. 0.5 using the TOXIper database^6^ (available until October 20th, 2020). Additional virulence factors such as sialidases, collagenases, and secondary toxins like *pfo*, *iam*, and *cpb2* were screened using the Virulence Factor Database (VFDB; March 17th, 2017; Chen et al., 2016). The Resfinder database (available until July 8th, 2017; Zankari et al., 2012) was used for the Antimicrobial Resistance (AMR) evaluation since it was the most complete database available with 2,228 AMR sequences at the moment of the analysis.

## Results

### Long-range dispersion of *Clostridium perfringens*

**Figure 1:**
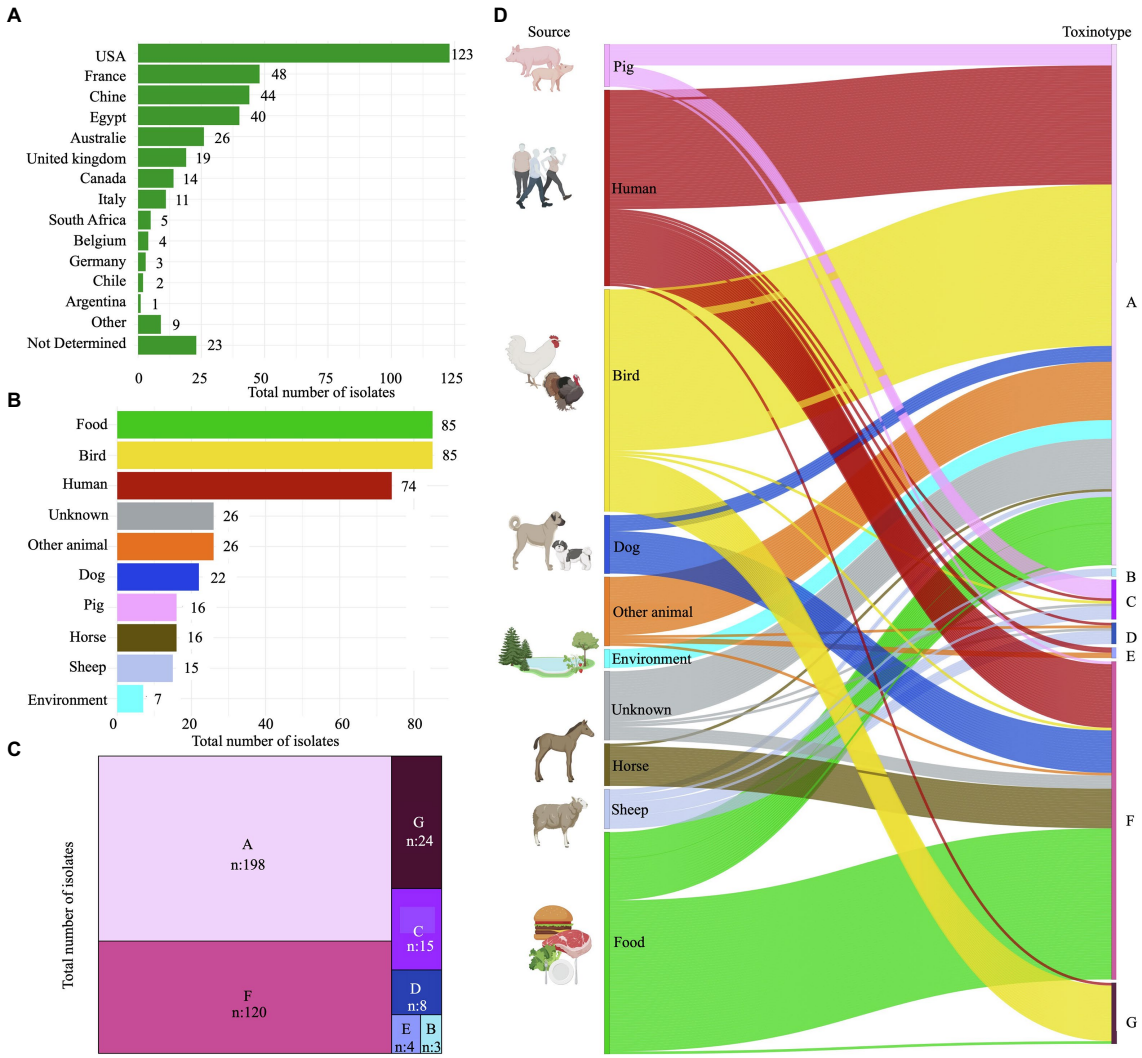
Origin and toxinotype profile of 372 *Clostridium perfringens* genomes. **(A)** Distribution of isolates by country of origin. **(B)** Distribution of isolates by source. **(C)** Distribution of isolates by toxin type. **(D)** Toxinotype diversity among hosts. All figures were created with R software.

A total of 372 genomes from different sources were collected for our study: two *de novo* sequenced genomes from Chile, 237 previously assembled sequences, and 133 raw reads (Supplementary Table S1). The number of contigs among the assemblies was variable, with an average of 82 contigs per genome. On overage, the genome size was 3.3 Mb, with a low GC content of around 28% (Supplementary Table S2). In addition, ANI values were invariably > 95% for the 372 genomes when compared to the reference strain *C. perfringens* ATCC 13124, confirming their classification as *C. perfringens* (Supplementary Figure S1). Most of the publicly available genomes were obtained from the US (123/372, 33.1%), France (48/372, 12.9%), and China (44/372, 11.8%; Figure 1A), and were recovered mainly from food (85/372, 22.8%), birds (85/372, 22.8%), and humans (74/372, 19.8%; Figure 1B).

### Wide toxinotype diversity among multiple hosts

The seven different toxinotypes defined until now were seen among our 372 isolates. Toxinotype A was the most frequently found (198/372, 53%), followed by toxinotype F (120/372, 32%), and G (24/372, 7%; Figure 1C). Interestingly, toxinotype distribution varied among hosts. In this regard, 60% (45/74) and 32% (24/74) of the human isolates were classified as toxinotype A and F, respectively. Two human isolates (2.7%) belonged to toxinotype E, and toxinotype C, D, and G had one (1.3%) isolate each. Food isolates were also distributed in a similar way, as 32% (27/85) were toxinotype A, 67% (57/85) F, and 1% (1/85) G.

As for animals, 72% (61/85) of the bird isolates were classified as toxinotype A, 26% (22/85) as G, and 1% as C (1/85) and F (1/85). Fifty percent of the swine isolates (8/16) corresponded to toxinotype A, 43.7% (7/16) to C, and 6.3% (1/16) to F. Likewise, the ovine isolates were classified as toxinotype A (2/15, 14%), B (3/15, 20%), C (5/15, 33%), and D (5/15, 33%). Isolates from canines (16/22, 73%) and equines (15/16, 94%) belonged to toxinotype F and only a few to toxinotype A (6/22, 27% and 1/16, 6%, respectively). The high diversity of toxinotypes in the multiple evaluated hosts is an indicator of heterogeneous toxins that can support the differential pathogenic effect of *C. perfringens* populations circulating in each of these species.

### *Clostridium perfringens* clones of heterogeneous origins

Multilocus sequence typing (MLST) was used to classify *C. perfringens* genomes. A total of 195 STs were identified among 322 genomes. One or more genes from the MLST scheme could not be recovered from 50 genomes, thus they were not assigned an ST. *cpa*, *colA*, and *nadA* were the genes with the most alleles (84, 84, and 73 respectively) compared to other MLST genes, which had less than 50 alleles (Supplementary Table S3).

**Figure 2:**
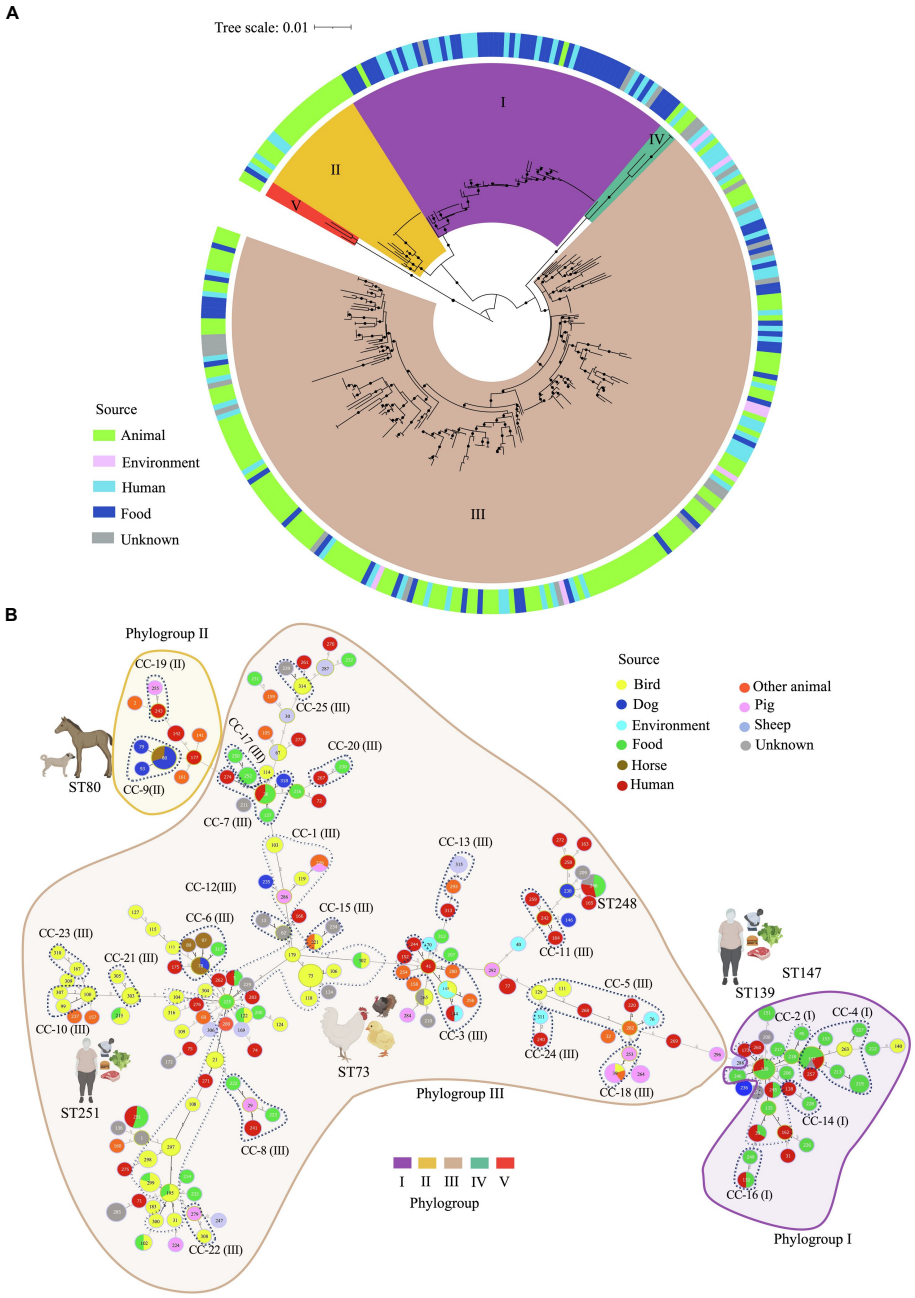
Multilocus sequence typing (MLST)-based phylogeny and goeBURST full minimum spanning tree of 195 *C. perfringens* MLST profiles among 322 genomes. **(A)** MLST-based phylogeny tree obtained with fastMLST. The outer ring shows the origin of the isolates as indicated in the legend. Clusters are represented by different colors on the inside of the ring. **(B)** MLST-based minimum spanning tree obtained with *goe*BURST. Sequence types (STs) are displayed as circles. Founder STs were defined as the STs with the greatest number of single-locus variants. Circle size indicates the number of isolates in every particular ST, with each color representing a different source type. Lines represent closely related isolates and line length illustrates STs that vary by one, two, or more alleles in their MLST profile. Clonal complexes (CC), defined as closely related STs that differ up to two of the eight loci used in the MLST scheme from a common founder (DLV, double locus variant), are designated by dashing lines.

MLST loci from the 322 genomes, were concatenated and aligned to generate a phylogenetic tree (Figure 2A), where five clusters were identified using the phylogenetic relationships and biological features as criteria, as reported before for *C. perfringens* (Abdel-Glil et al., 2021) Overall, Cluster III isolates were more frequent in our dataset (226/322, 70%). This cluster grouped isolates from diverse sources, including animals (120/226, 53%), humans (44/226, 20%), food (34/226, 15%), unknown sources (21/226, 9%), and environment (21/226, 3%). Cluster I (67/322, 20%) grouped mostly isolates from food (42/67, 63%) along with isolates from human (19/67, 28%), animal (3/67, 4.5%), and unknown sources (3/67, 4.5%). Interestingly, Cluster II (23/322, 7%) only grouped isolates from animals (19/23, 83%) and humans (4/23, 17%), whereas Cluster IV was formed by 3 isolates (3/322, 1%) from animal origin, and Cluster V by 3 isolates (3/322, 1%) from animal (2/3, 66%) and food (1/3, 34%) sources (Figure 2A).

Of the 195 STs detected, five were the most common: ST-147, -248, -80, -251, and ST-73 (Supplementary Figure S2). Isolates within these STs tended to have the same toxinotype, however, they were located across different clusters. For example, 14 toxinotype F isolates from food and human sources from the US and Italy isolated during 2017 and 2019 were classified as ST-147 and grouped in Cluster I. In the same way, 13 toxinotype F isolates from dogs and horses from Canada and the US, were classified as ST-80 located in Cluster II. Furthermore, ST-251 included 11 isolates from toxinotype F in cluster I recovered in the US. In contrast, ST-73 grouped 11 isolates from toxinotypes G and A from birds, all of them grouped in Cluster III.

The MLST-based minimum spanning tree built using the *goe*BURST algorithm led to evolutionary inferences. By identifying founder STs in the 25 CC and 95 singletons (Figure 2B; Supplementary Figure S3). Overall, CC1 was the most commonly represented CC. It was found within Cluster III, grouping 23 STs from 50 genomes mostly from birds (ST-73, -106, -118), human stool and blood samples (ST-5, -262 y ST-271), and food (ST-5, -195, -225, -299 y ST-302). The founder ST for this group was ST-225, which is associated with food. Usually, the predicted founder corresponds to the most predominant ST in a CC (Feil et al., 2004), however this ST contained only one isolate whereas DLV descendant ST-73 contained 11 isolates (Supplementary Figure S3). This interesting finding might be caused by an origin bias due to either the relatively low number of samples used or by the natural selection pressure within the population leading to the emergence of strains with a strong adaptive advantage (Feil et al., 2004).

Most of the isolates from human stools and food were grouped in Cluster I as CC-2 along with ST-139 as founder ST. ST-39 and ST-253 from swine farms in China were classified as CC-18 in Cluster III, and founder ST-80, -93, and -79 from canine and equine isolates were in cluster II as CC-9. Some of the STs varied in terms of hosts, for instance ST-80 and ST-78 included isolates from dogs and horses, ST-5, -33, -132, -139, -147, -248, and -251 included human and food isolates, and ST-39 grouped isolates from swine and birds, while ST-143 grouped birds and environment, and ST-215 birds and food (Figure 2B).

### Five *Clostridium perfringens* phylogroups identified by core genome-based phylogenetic analysis

**Figure 3:**
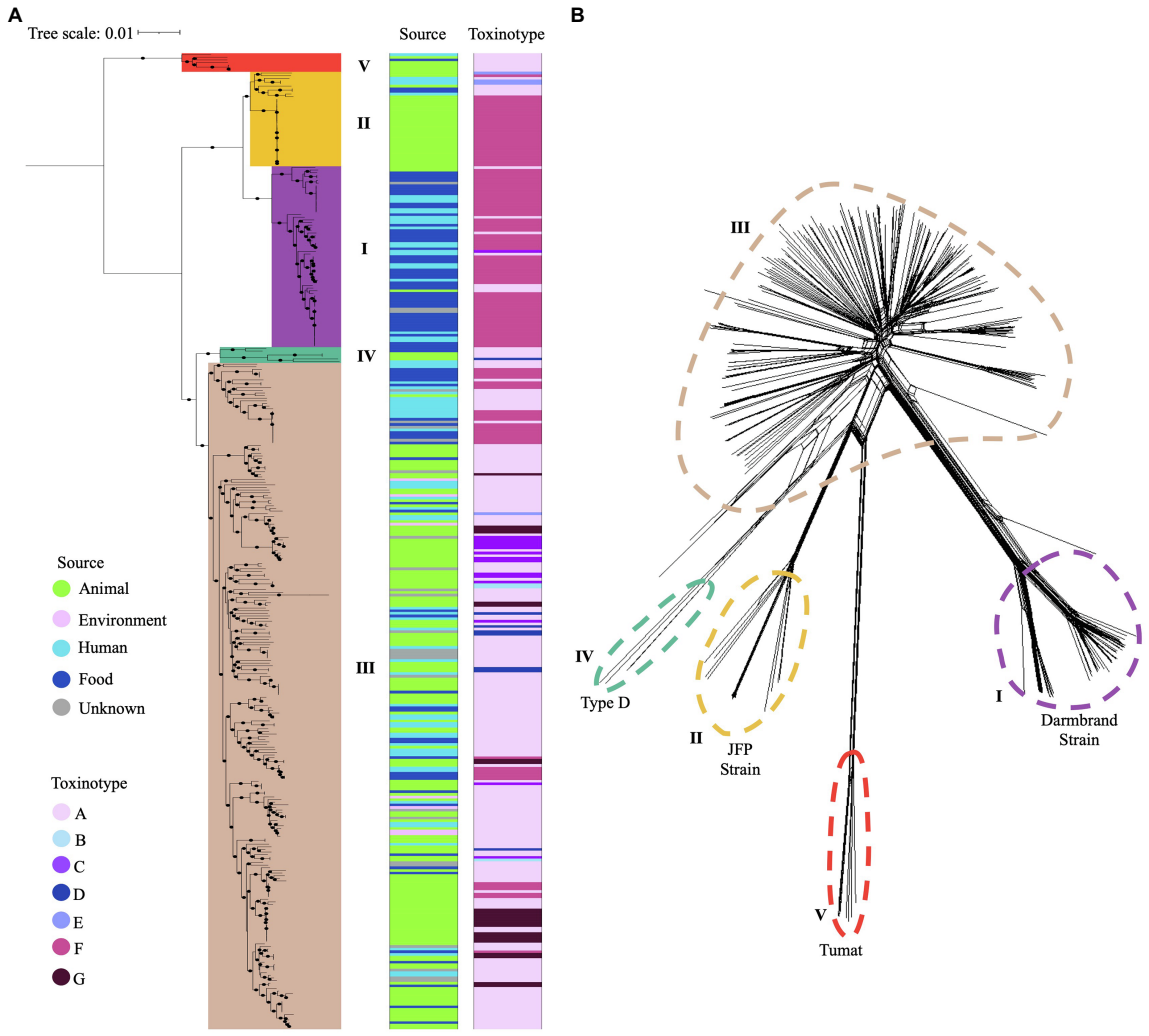
Phylogenetic grouping of *C. perfringens* using two different approaches. **(A)** Phylogenetic tree based on the core genome of 372 genomes. Five main phylogroups are highlighted in different colors. Source type and toxinotype are shown on the right panels. **(B)** Five phylogenetic networks based on the Neighbor-Joining (NJ) algorithm are shown with different colors.

A total of 35,876 genes are included in the pangenome of this dataset. Of them, 34,917 (97.3%) genes are accessory and only 959 (2.7%) are considered part of the core genome. In the resulting tree, five main branches were observed matching the clusters that were established by using the reference genomes and a bootstrap ≥ 80% (Figure 3A). The core genome-based phylogenetic tree matches with the MLST-based topology, as well as with the phylogenetic network generated using neighbor-joining, confirming the classification of *C. perfringens* population in five distinct clusters as reported previously (Figure 3B).

Phylogroup I (*n* = 69) clustered most of the isolates from humans and food from toxinotype F (*n* = 57) that were *cpa* and *cpe* positive, along with some isolates from toxinotype A (*n* = 7) from humans and animals. Phylogroup II (*n* = 36) grouped canine and equine isolates from toxinotype F (*n* = 26), as well as one cattle and two human isolates from toxinotype E. Phylogroup III (*n* = 257) was the most diverse group, where the novel isolates recovered from water and a human in Chile were located. In this phylogroup isolates from birds, food, environment, and humans classified as toxinotype A (*n* = 177), toxinotype F (*n* = 31), toxinotype G (*n* = 24), and in less proportion toxinotype C (*n* = 14), D (*n* = 7), and B (*n* = 3) were also found. Phylogroup IV (*n* = 3) and V (*n* = 7) included a majority of toxinotype A isolates (Figure 3A).

### *Clostridium perfringens* is a highly versatile pathogen with a large number of virulence factors

**Figure 4:**
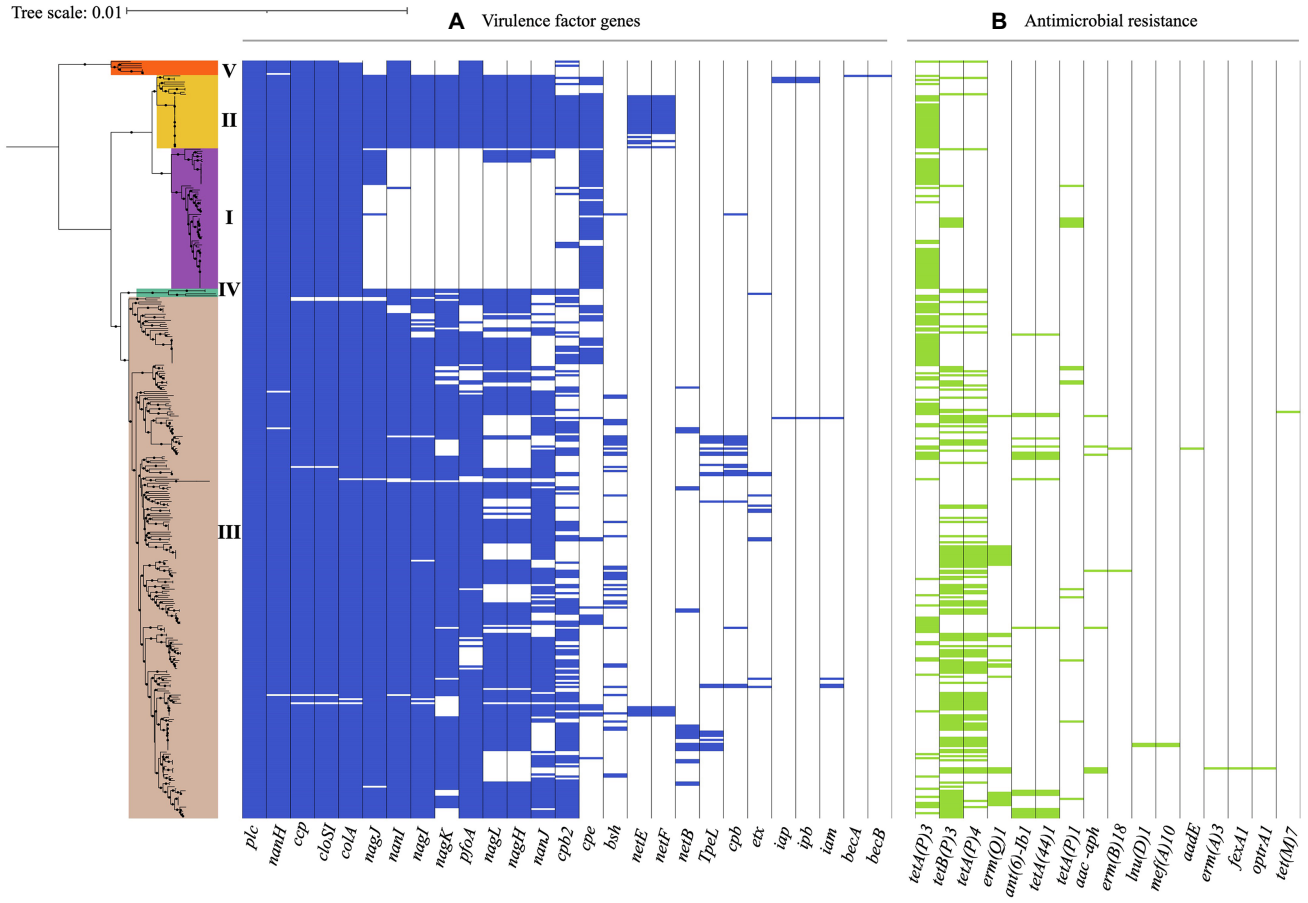
Virulence-associated and antimicrobial resistance (AMR) genes in 372 *C. perfringens* genomes. The core genome-based phylogenetic tree showed phylogroups highlighted in different colors. Matrix of absence and presence of genes shows **(A)** virulence genes **(B)** antimicrobial resistance (AMR) genes. The minimum coverage threshold needed for detection of these genes was 70% and the percentage of identity was 99.95%. The resulting trees were visualized and edited using iTOL v.5 (Letunic and Bork, 2021).

A total of 372 WGS were analyzed to evaluate the distribution of virulence genes. The alpha-toxin gene *plc* associated with gangrene in humans and several animals, and possibly involved in enterotoxemia and gastrointestinal disease in ruminants, horses, and swine was present in most of the isolates, along with genes encoding extracellular enzymes such as alpha-clostripain (*ccp*), hyaluronidases (*nagH*, *nagI* and *nagJ*), and alpha-clostripain collagenase (*colA*), as expected (Canard et al., 1994; Sakurai et al., 2004; Goossens et al., 2017; Geier et al., 2021). Other genes present in the majority of the isolates were *pfoA* (perfringolysin O), *tpeL* (toxin perfringens large), and *cpb2* (beta2 toxin), which are protein coding genes involved in gastrointestinal outcomes and gangrene (Coursodon et al., 2012; Bueschel et al., 2013; Chen and McClane, 2015), and the sialidases *nanH*, *nanI* y *nanJ*, which play an important role in colonization and immunomodulation (Figure 4A).

The presence of virulence genes varied among the phylogroups. For example, all of the isolates in phylogroup I carried *nanH*, however *nanI, nanJ*, *nagH*, *pfoA,* and *cpb2* were absent in this group, in contrast to phylogroup II, where these genes were found in all of the isolates along with *nagI* and *nagK*. Additionally, 64% (23/36) of the isolates in this phylogroup also carried *netE* and 58% (21/36) *netF*. While the presence of these virulence genes in phylogroup III was variable, this was the only group where *TpeL*, a member of the large clostridial toxin (LCT) family involved in cell cytotoxicity was present, especially in isolates of toxinotype A, B, C, and G. However, this gene was not detected in type D or F isolates carrying the *cpe* and *itx* toxin genes. This difference in toxin profiles can be attributed to a potential incompatibility between plasmids carrying these genes (Chen and McClane, 2015).

Isolates in phylogroup IV carried the coding genes for the hyaluronidases *nagH*, *nagI,* and *nagJ*, as well as *nanI* and *pfoA*. Phylogroup V, which cluster toxinotype A isolates carried *nanI* and *pfoA*, unlike the toxinotype A isolates in phylogroup I (Figure 4A). These findings suggest that the differential presence of virulence factors in phylogroups may be due to selective advantage conferred by determinants in different niches (Sawires and Songer, 2006), routine treatment with clostridial toxoids especially in ruminants, or even environmental differences in the geographical regions (Simpson et al., 2018).

### Prevalence of antibiotic resistance genes in *Clostridium perfringens*

AMR gene were found in 72.8% (271/372) of the genomes, with *tetA* (107/271, 39.5%) and *tetB* (98/271, 36.2%) involved in tetracycline resistance, being the most frequent and commonly found in phylogroup III isolates (Figure 4B). Interestingly, the water isolate from Chile harbors genes for tetracycline resistance (*tetA*), while the one from human stool possesses genes that encoded tetracycline (*tetA, tet44*) and aminoglycoside resistance (*ant(6)-Ib1*). Likewise, this approach identified genomes carrying *ermQ*, (28/372, 7.5%) an erythromycin resistance methylase gene mainly found in birds and environment isolates grouped in phylogroup III and classified as toxinotype A. *ant(6)-Ib1* genes encoding aminoglycoside resistance were found in some toxinotype A and C isolates from swine and birds from phylogroup III (Figure 4B).

## Discussion

*Clostridium perfringens* is a clinically relevant pathogen due to its presence across several host species and its capacity to cause numerous medically important intestinal diseases in humans and animals (Mehdizadeh Gohari et al., 2021). To assess the genetic variation within the species, as well as to establish the phylogenetic relatedness, we collected the publicly available genomes of 370 isolates collected between 2010 and 2020 from different countries, with a majority of the isolates obtained in developed countries such as the US, France, and China (Figure 1A). There is a poor representation of isolates from developing countries in our dataset, possibly due to limited epidemiologic surveillance and genomic data collection, especially in South America, from where only one genome from Argentina was found and two more from Chile were added in this study.

The dataset also has a high percentage of strains isolated from stool samples of animals used for human consumption as well as isolates from food origin (Figures 1B, 2B), which along with the evidence of food products such as milk, meat, poultry, and pork among others as a source of infection, confirm the key role that *C. perfringens* plays as one of the most common causes of foodborne illness (Brynestad et al., 1997; Grass et al., 2013; Bintsis, 2017; Xu et al., 2021). However, despite the high percentage of isolates of human and food origin, it should be noted that environmental or commensal isolates are scarce (Figure 1B). In addition, some metadata variables are unknown, such as the possible association of genomes with outbreaks, which could underestimate the diversity of *C. perfringens* and affect the results of AMR prevalence, emphasizing the need for further studies with a greater number of genomes.

*Clostridium perfringens* toxinotypes are associated with heterogeneous diseases such as clostridial myonecrosis (gas gangrene) or gastrointestinal infections in humans and animals (Rood et al., 2018). Toxinotype G, associated with necrotic enteritis and gangrenous dermatitis in birds (Yang et al., 2019; Kiu et al., 2019a), and toxinotypes D and E that cause illness in sheep and cattle (Layana et al., 2006; Uzal and Songer, 2008; Nazki et al., 2017; Rood et al., 2018), have not been described in humans before, however, we identified one isolate from a raw chicken patty and another from human blood as toxinotype G (Figure 1D). Likewise, we found toxinotype D and E strains isolated from human stools. These findings support previous studies that have reported *C. perfringens* type D and E strains harboring *etx* and *iap* in humans (Al-Shukri et al., 2021), which can explain potential routes of transmission in subjects that have been in contact with infected animals or have consumed contaminated food (Songer, 2010; Kiu et al., 2019b). Furthermore, these findings might be related to *C. perfringens* strains considered as normal intestinal microbiota that are in contact with acquired strains carrying conjugative plasmids that are often associated with insertion sequences that can mobilize toxin genes between different strains. This could lead to the conversion into virulent toxin-producing strains and the emergence of specific toxinotypes in new hosts (Freedman et al., 2015). Future studies should include genomes assembled with a standard pipeline that includes an approach to recover extrachromosomal elements in order to describe the plasmidome of *C. perfringens* and thus contribute to the biological knowledge of this species.

MLST and geoBURST analyses have not been widely used for *C. perfringens*: However, the identification of the founder STs, that differs from other STs at only one locus, provide a tool for epidemiological and evolutionary investigations of emergent pathogens (Feil and Enright, 2004). Furthermore, the identification of 25 CC in this work allowed us to compare the distribution of *C. perfringens* isolates from animals, humans, food and environment (Figure 2B; Supplementary Figure S2), evidencing a close relationship between isolates from different hosts. These findings support the strong association with foodborne diseases and suggesting their zoonotic potential and high diversification of this specie, as previously described (Verma et al., 2020; Hassani et al., 2022; Xiu et al., 2022).

The CC including toxinotype G isolates from human and birds, and the evidence of the distribution of human, food, and animal isolates in the same ST or CC matches the zoonotic potential of *C. perfringens* as demonstrated by other published studies (Immerseel et al., 2004; Mwangi et al., 2019; Verma et al., 2020). Population structure analyses based on MLST (Figure 2A) revealed five clusters in line with those generated here using the core genome (Figures 3A,B), and with results from previous studies (Abdel-Glil et al., 2021). Although MLST has a limited ability to establish phylogenetic relations since it only uses fragments from eight housekeeping genes, it is still a useful tool for interspecies *C. perfringens* typing due to its reproducibility, high discriminatory power, and easy accessibility (Pérez-Losada et al., 2013; Jolley et al., 2018; Guerrero-Araya et al., 2020). Despite these advantages, WGS has emerged as a more robust and complete tool contributing to the investigation of phylogenetic relatedness among isolates and allowing a deeper understanding of transmission dynamics, emerging clones, key virulence loci, and the presence of AMR genes (Salipante et al., 2015; Quainoo et al., 2017; Pightling et al., 2018).

The pangenome analysis of *C. perfringens* conducted in this study showed an accessory genome of 97.32%, an extremely high percentage in comparison with other closely related bacteria such as *C. paraputrificum*, species with an accessory genome of 67%, *C. tertium* with 37.6% of accessory genes (Muñoz et al., 2019), or *C. baratii* with an accessory genome of 24.43% (Silva-Andrade et al., 2022). This high level of genome plasticity is similar to the one found in *Clostridioides difficile*, which has an accessory genome of 87.2% (Knight et al., 2021). *C. difficile* and *C. perfringens* are part of the normal intestinal flora but can become gastrointestinal pathogens, which can be explained by the ability to express different phenotypes in response to particular environmental conditions. The high genome plasticity of *C. perfringens* can give rise to the emergence of populations carrying new toxigenic profiles by acquired virulence factors due to horizontal gene transfer (HGT) leading to rapid genetic evolution. Thus, this genomic plasticity of *C. perfringens* is a determinant in the adaption to different hosts, as well as in the increase of its pathogenic potential and survival in different environments (Brüggemann, 2005).

The importance of the acquisition of different virulence factors in *C. perfringens* is given by their adaptation within different disease niches. When exploring the virulence factors present in the *C. perfringens* genomes, we found that *plc, colA,* and *nanH* are present in the majority of the genomes, hence they cannot be considered markers with high host discrimination capacity (Goossens et al., 2017; Mahamat Abdelrahim et al., 2019). The identification of isolates carrying *netE* and *netF* toxins in two animal species (dogs and foals) of phylogroup I, as well as previous reports of the prevalence of these two toxins in *C. perfringens* isolates from dogs with acute diarrhea hemorrhagic syndrome (Sindern et al., 2019), suggest their adaptability to these specific hosts. Likewise, the presence of three sialidases (*nanH*, *nanI*, and *nanJ*) in isolates from the same clinical outcomes highlights the role of these enzymes in increasing the adhesion of *C. perfringens* to host cells (Carman, 1997; Chiarezza et al., 2009; Li and McClane, 2021) and suggests an important role in the intestinal pathogenesis in these hosts, as was previously reported (Li et al., 2015; Li and McClane, 2021). However, a better understanding of the role and specificity of these molecules in canine acute hemorrhagic diarrhea and necrotizing enteritis of foals is required.

Another virulence-related gene, *pfoA*, was also found in most of the genomes included here (Figure 4A), however, it was absent in toxinotype F isolates clustered in the phylogroup I, which correlates with prior studies that have revealed that some strains that produce enterotoxins and therefor cause food poisoning lack *pfoA* (Myers et al., 2006; Deguchi et al., 2009). This suggests that this cytolytic toxin is not the main cause of most gastrointestinal outcomes in humans, however, it could be associated with the enteritis necro-hemorrhagic or bovine enterotoxemia, as recent studies have shown (Verherstraeten et al., 2013a,b). Thus, the high number of virulence-related genes in the 372 genomes analyzed, especially in the novel isolates from Chile included in our study (Figure 4A), reveals the need to continue with the epidemiological surveillance and the molecular study of virulence factors, mainly in unexplored countries, to provide more data for a deeper knowledge of the global diversity of *C. perfringens*.

Another group of molecular markers of importance in health is the one associated with antibiotic resistance. We found that a high percentage of the genomes (72.8%, 270/372) harbored some type of AMR gene (Figure 4B), where a large number of isolates carried genes putatively linked to resistance to tetracycline, macrolides, and aminoglycosides. The presence of a high number of AMR genes in isolates from pigs and birds could be a consequence of the use of antibiotics as growth promoters in these animals and may be related to the appearance of resistance in zoonotic pathogens (Osman and Elhariri, 2013). Although WHO has questioned the use of additives due to the risk of antibiotic residues in meat products for human consumption, several countries continue with this practice that can be related to the increase of antibiotic resistant strains (Salvarani et al., 2012). Many factors such as the production of high concentrations of antibiotics in the global industry (Nijsingh et al., 2019; Tell et al., 2019), the indiscriminate use of antibiotics in the community (Graham et al., 2019), the contamination of natural sources by hospital waste (Larsson and Flach, 2021), the impact of intrahospital infections caused by multidrug resistant strains (Avershina et al., 2021), the use of antibiotics for animal growth, the poor management of organic waste and the use of animal excrement in the agricultural sector (Tang et al., 2017), and the antimicrobial drugs overused during the first wave of COVID-19 (Manesh and Varghese, 2021), pose a high risk for possible pathways of antimicrobial resistance (Chokshi et al., 2019).

The analysis of 372 genomes conducted here, is the largest effort to snapshot the global genomic diversity of *C. perfringens* to date. The genomic plasticity of this microorganism due to its low GC content (~ 28%; Uzal et al., 2014), its short doubling time (~ 7 min; Maiden, 2006), and a high percentage of horizontally transferred toxin encoding genes (Xiao et al., 2012; Uzal et al., 2014) contributes to the spread of existing toxinotypes to new hosts, as well as to the increase of food poisoning outbreaks and the growth of AMR. The use of MLST and WGS in a “One Health” framework that connects the health of humans and animals in a shared environment represents an optimal approach to advance knowledge of the global genetic diversity of *C. perfringens.* Our findings emphasize the need for further studies using a larger number of isolates from different ecological niches to elucidate the genetic characteristics, diversity, and zoonotic potential of *C. perfringens* and to improve strategies to reduce the growing threat to public health by this pathogen.

## Data availability statement

The datasets presented in this study can be found in online repositories. The names of the repository/repositories and accession number(s) can be found in the article/Supplementary Material.

## Author contributions

AC, EG-A, and MM designed the study and performed the data analyses. AC and MM performed methodology, formal analysis, data curation, and visualization and wrote the original draft preparation. JDR, DP-S, CR, SC, LV, and MC-A validated the results and revised and edited the manuscript. JDR and MM supervised the study and contributed to review and editing the manuscript. All authors contributed to the article and approved the submitted version.

## Funding

This study was financially supported by Dirección de Investigación e Innovación from Universidad del Rosario, Bogotá D.C. and the Universidad de Boyacá, Tunja, Colombia. In addition, this work mas supported by start-up funds from Texas A&M University and by ANID, Millennium Science Initiative Program–NCN17_093 to DP-S.

## Conflict of interest

The authors declare that the research was conducted in the absence of any commercial or financial relationships that could be construed as a potential conflict of interest.

## Publisher’s note

All claims expressed in this article are solely those of the authors and do not necessarily represent those of their affiliated organizations, or those of the publisher, the editors and the reviewers. Any product that may be evaluated in this article, or claim that may be made by its manufacturer, is not guaranteed or endorsed by the publisher.

## Footnotes

^1^ https://www.ebi.ac.uk/ena/browser/home

^2^ http://www.bioinformatics.babraham.ac.uk/projects/fastqc/

^3^ https://github.com/widdowquinn/pyani

^4^ https://github.com/EnzoAndree/FastMLST

^5^ http://goeburst.phyloviz.net

^6^ https://github.com/raymondkiu/TOXIper
