# Supplementary material for: Intra-species diversity of Clostridium perfringens: A diverse genetic repertoire reveals its pathogenic potential
Source: Front Microbiol. 2022 Jul 22;13:952081. doi: 10.3389/fmicb.2022.952081 (PMC9354469; doi:10.3389/fmicb.2022.952081)
Supplement: Supplementary file 2 [file Data_Sheet_1.PDF]

Supplementary Material

Intra-species diversity of *Clostridium perfringens*: a diverse genetic repertoire reveals an emerging pathogenic potential

Anny Camargo[1,2], Enzo Guerrero-Araya[3], Sergio Castañeda[1], Laura Vega[1], María X. Cardenas-Alvarez[1,4], César Rodríguez[5], Daniel Paredes-Sabja[3,6], Juan David Rámirez[1,7] and Marina Muñoz[1,3*].

[1] Centro de Investigaciones en Microbiología y Biotecnología-UR (CIMBIUR), Facultad de Ciencias Naturales, Universidad del Rosario, Bogotá, Colombia.

[2] Health Sciences Faculty, Universidad de Boyacá, Tunja, Colombia.

[3] ANID – Millennium Science Initiative Program - Millennium Nucleus in the Biology of the Intestinal Microbiota, Santiago, Chile.

[4] Department of Pharmacology, University of North Carolina, Chapel Hill, North Carolina, United States.

[5] Centro de Investigación en Enfermedades Tropicales, Universidad de Costa Rica, San José, Costa Rica; Laboratorio de Investigación en Bacteriología Anaerobia, Facultad de Microbiología, Universidad de Costa Rica, San José, Costa Rica.

[6] Department of Biology, Texas A&M University, College Station, Texas, United States.

[7] Molecular Microbiology Laboratory, Department of Pathology, Molecular and Cell-Based Medicine, Icahn School of Medicine at Mount Sinai, New York, NY 10029, USA

Supplementary Figures

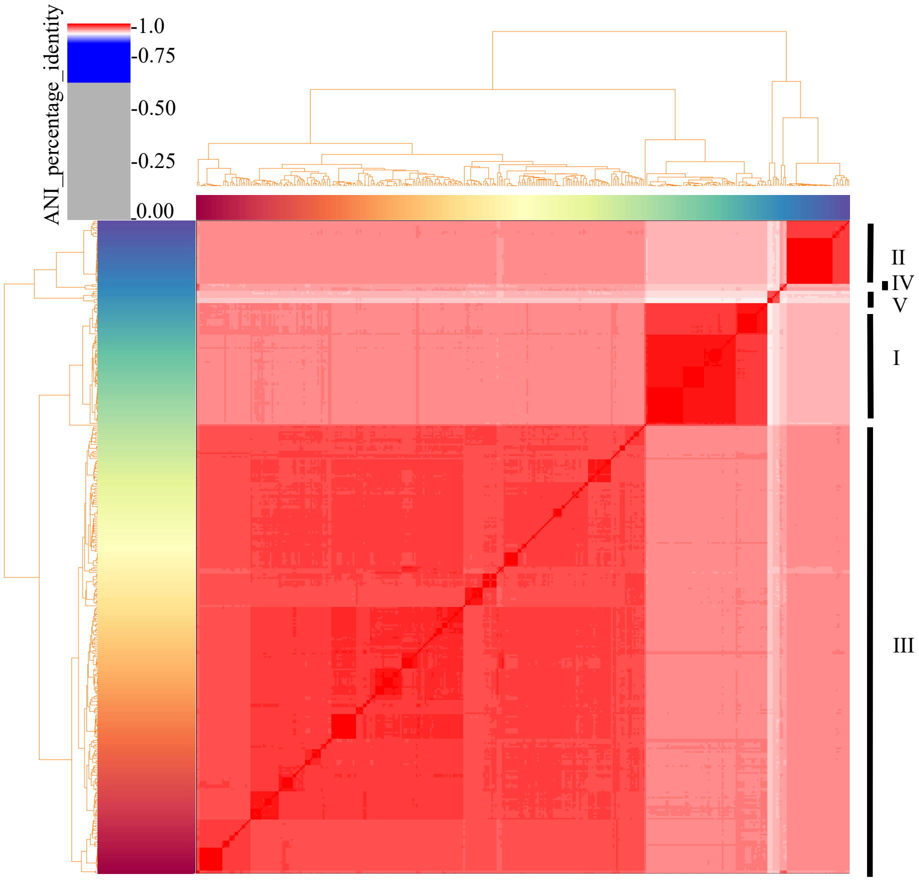

Supplementary Figure S1. Average nucleotide identity (ANI) analysis. Pairwise comparison of 372 *C. perfringens* genomes using pyANI. Percentages greater than 95% indicate that the isolates belong to the same microbial species. The corresponding phylogroups are indicated on the right panel.

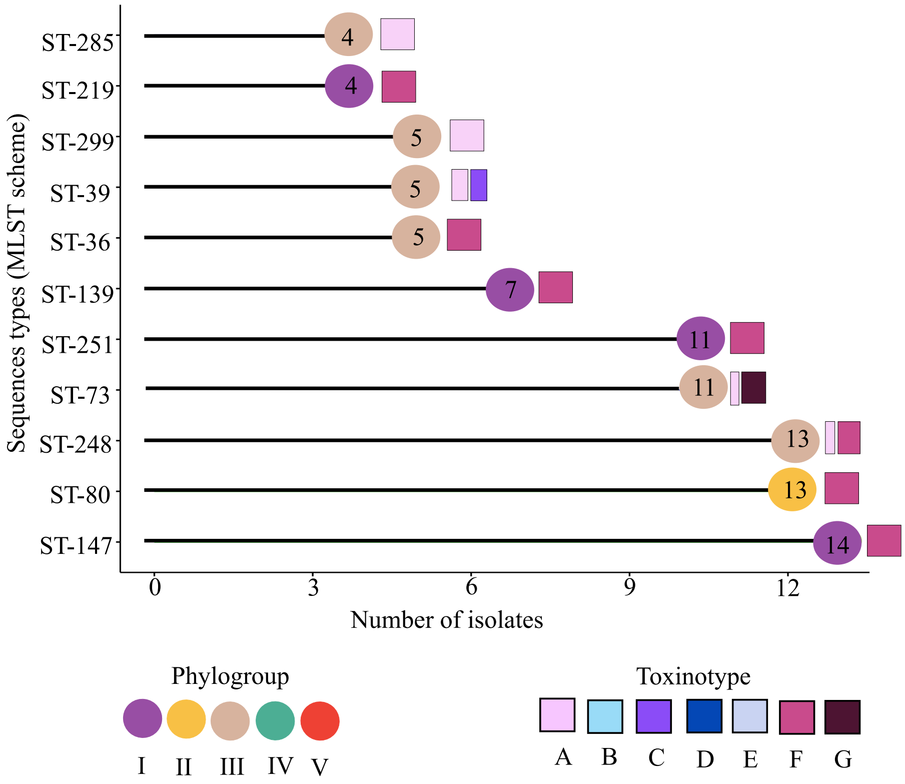

Supplementary Figure S2. Number of isolates assigned to sequence type (ST) using the MLST scheme. The number of isolates is denoted by the number in each circle. Phylogroups are designated by the circle color. Toxinotypes are indicated by square colors. The figure only includes STs with a number of isolates ≥ 4.

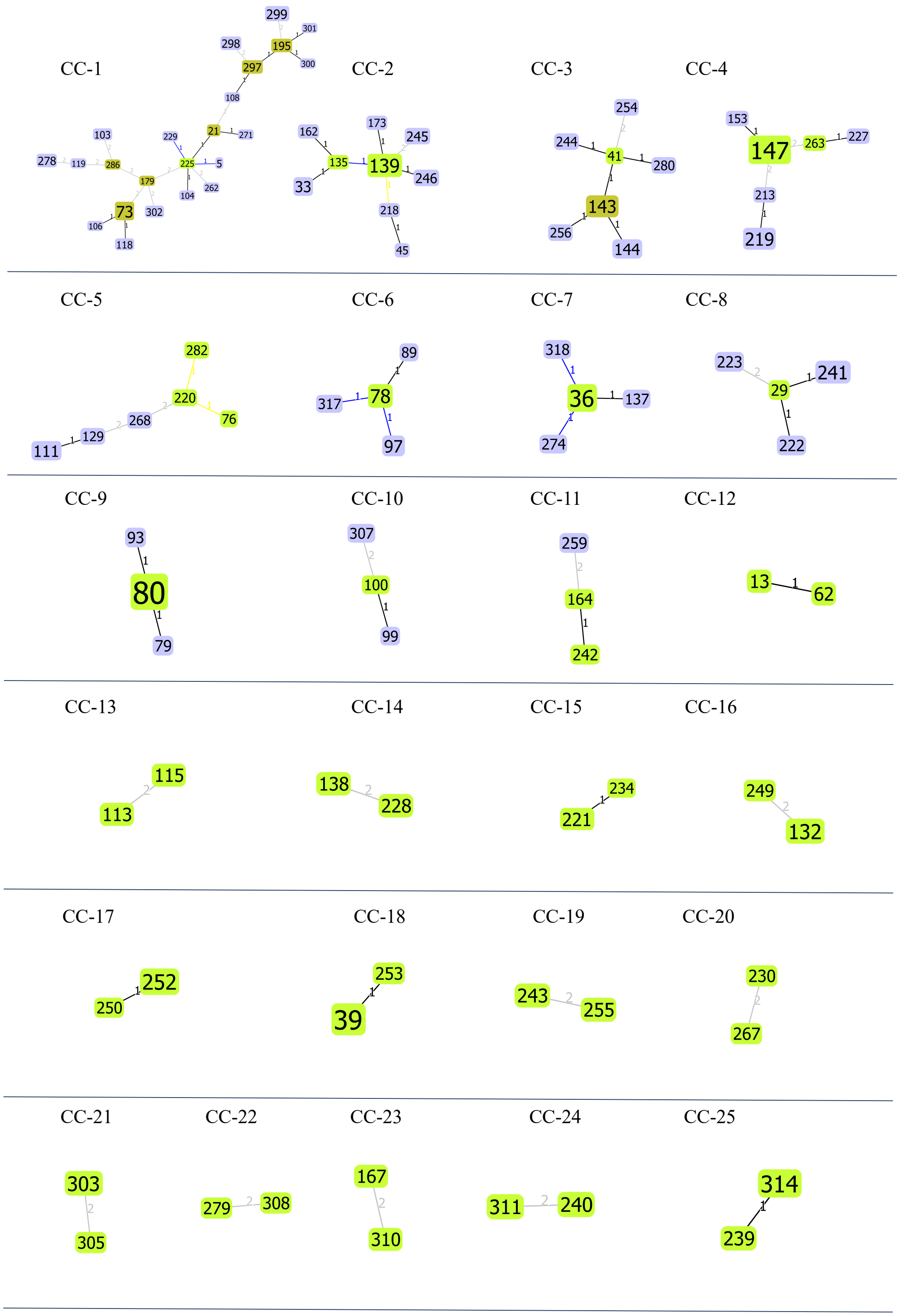

Supplementary Figure S3. Diagram of the main clonal complexes identified by goeBURST analysis. Graphical representation of the clonal complexes and its founder ST. Light green represents the founder ST and dark green the subgroup founder.

Supplementary Tables

Supplementary Table S1: Metadata of the 372 *C. perfringens* strains included in the study (excel file - sheet ‘Table S1’).

Supplementary Table S2: Quast information: genomes assemblies of *C. perfringens* strains (excel file - sheet ‘Table S2’).

Supplementary Table S3: MLST information of the 372 *C. perfringens* strains included in the study (excel file – sheet ‘Table S3’).
